# Radiant Light and Heat

**Published:** 1923-07

**Authors:** Wm. Benham Snow


### RADIANT LIGHT AND HEAT

BY WM. BENHAM SNOW, M. D.

*Penetration:* It is generally conceded that the penetration is inversely as the frequency and directly as the wave length. In other words, the lower frequencies, having a corresponding increase in wave length penetrate relatively deeper than the high frequencies. It is generally recognized that the ultra-violet radiations do not penetrate more than one and one-half to two milliameters into the tissues and are, therefore, inefficient in their destructive effects upon germ life except near the surface, where they exert the greatest energy upon the superficial layers of the skin, with prolonged exposures producing profound blistering; the toleration to lengthening exposures increasing during a series.

It is always observed when viewing the penetration of light, when an electric light is passed through the cheek from within the mouth or through the hand that the red rays are the ones that come through in great abundance and that the yellow rays do not appear. The infra-red or heat rays are just below the red, and naturally though not visible and therefore not demonstrable, resemble them in other characteristics with a longer wave length and naturally penetrate deeper, increasing in penetration as the colored rays have from the ultra-violet to the red. The infra-red represent from the sun's rays eighty per cent. of the intensity of the sun's energy and fifty per cent. of the intensity of the mercury vapor lamp and are probably to be considered the most important factor in the therapeutics of radiant energy from luminous sources in the production of hyperemia by conversive heat except the superficial effect of the ultra-violet rays as they come of the sources of radiant energy.

As the red rays penetrate deeper there is no occasion to doubt that the infra-red rays penetrate much deeper than the visible rays. The infra-red rays penetrate deeper into the tissues and have a greater therapeutic effect in the deeper tissues which alone will explain the curative results in cases of cholecystitis, early appendicitis and other intra-abdominal conditions to which depths the visible rays are demonstrated not to penetrate.

As a result of the experimental work of Dr. Virgil C. Kinney we now have the following contribution to this report, he having shown the effects as to penetration by careful, scientific tests.

(1) Natural sunlight has a greater depth of penetration, as far as experiments indicate, than any known artificial form of radiant energy with the exception of radium and the X-rays. (2) Fluoroscopic or ocular tests show that light—the visible spectrum—will penetrate living animal tissues in great quantities from one-half inch or less up to $1\frac{1}{8}$ inches. An opaque body passed between the light and the exposed part produces a distinct shadow. From $1\frac{1}{8}$ inches up to $1\frac{3}{8}$ inches light comes through in a much lesser degree and is so faint that when an opaque body is moved between the exposed surfaces the light does not produce a shadow. Between $1\frac{3}{8}$ inches and $1\frac{7}{16}$ inches is an area admitting some light but of so much lesser degree, that it may be left out of calculation. At $1\frac{1}{2}$ inches practically all light is absorbed in transit, and, no matter how long the observation is carried on, nothing but darkness is encountered.

These same results have been obtained by means of very rapid partly achromatic photographic plates. Thus $1\frac{1}{2}$ inches of animal tissue is the greatest possible depth through which light will pass. (3) A pigmented skin offers no more resistance to the penetration of light rays than does a perfectly white skin when observed fluoroscopically or ocularly, while a photographic plate which theoretically is more sensitive to light rays than the human eye goes a step farther and shows that pigmented skin transmits light better than an unpigmented one. This he has repeatedly demonstrated by means of experiments with blondes, brunettes and negroes. This he attributes to the fact, that the pigment in a tanned skin, of a natural brunette, or a negro, which is brown, on exposure to light, reacts as a yellow color, and yellow transmits light better than any other color. (4) The pigment which comes into the skin because of contact with the ultra-violet of the sun's rays acts to increase the resistance against the burning and blistering effects of those rays. Mucous membranes pigment but little, if any, and so do not toughen and therefore are very prone to burning, cracking open and to sores. The human skin, if allowed to pigment, toughens and then will resist not only the irritating action of the sunlight, but also of strong winds and to a great degree the X-ray.

Dr. H. F. Pitcher reports on the ultra-violet rays as follows:

To Finsen belongs the honor of first making use in therapeutics of the chemical qualities of sunlight and its bacterial action, he invented a lamp which filters out the luminous and heat rays leaving the ultra-violet ray with which he revolutionized the treatment of tuberculosis of the skin.

Rollier, recognizing the actinic qualities of sunlight, treated successfully many cases of tuberculosis of the bones and joints of children by unfiltered sunlight in the high altitudes of the Switzerland mountains.

On account of the dust and smoke and various gases which lie close to the earth's surface in the lower altitudes, the ultra-violet rays are nearly filtered out, leaving the red rays which are heat rays.

Peter Cooper Hewett invented the mercury vapor lamp which was at first rather a crude affair, but produced large quantities of ultra-violet rays. Prof. Kromayer later remodeled and refined this lamp with a quartz tube practically what is used at the present time. Lamps of this character radiate rays rich in ultra-violet.

A vast amount of research has been expended upon radiant energy during the past century. There are two types of lamps—air cooled and water cooled—in use at the present time, both types received their ultra-violet energy from quartz mercury arc burners, but their physical construction and therapeutic applications are different. Investigators have found that the air-cooled lamp emits the longer waves (between 4,000 and 3,000 angstrom units, the water-cooled between 3,000 and 2,000 angstrom units); the air-cooled lamp rays are more penetrating than the short rays. They produce an erythema more quickly and with greater intensity. They are chemically oxidizing, and stimulating to metalbolim. The are biologic in character, while the shorter waves are to a greater degree bactericidal.

The air-cooled lamp is employed as a builder, while both are used to destroy bacteria. The water-cooled lamp destroys them with greater certainty. The long rays of the air-cooled lamp rapidly produce a hyperemia, and are absorbed into the capillary blood streams and carried to the different parts of the body, where they stimulate the phagocytic action of the leucocytes, and endothelial, leading to the formation of anti-bodies.

The absorption of the ultra-violet rays into the system, and there eradicating disease, can be better understood when we consider that the sun's radiant energy is the promoter of all life.

*Physiological Effects:* Taning is an effect upon the human skin which may be considered either a physical effect or physiological. This is due almost entirely to the higher frequencies of the ultra-violet radiation and possibly to a less degree of the violet. This is demonstrated by the absence of tanning when the ultra-violet rays are filtered out by passing the radiation through glass. Tanning may be looked upon as one of the fortunate natural provisions, whereby the individual exposed to the intense unfiltered ray of the sun is provided by this means with a protection against the extremely irritating effects. The black skin of the mulatto and the dark skin of the brunettes and the races which have had for centuries their habitat in the torrid and tropical regions of the earth are protected against these rays.

The production of hyperemia in the tissues is due to the physiological response of the vasomotor, or the reflex nervous mechanism to heat produced in the tissues by transformation of radiant energy into heat units. The establishment of increased circulation of blood in a part is provided to carry away the excess heat—a natural effect to maintain an equilibrium of temperature, and prevent injury to the tissues.

The physiological effects of radiant light and heat may be divided properly into (1) the effects upon general metabolism; (2) the local effects of stimulation upon the tissues; (3) to chemical and biological charges in the body's fluids, and (4) bactericidal effects.

The effects upon general metabolism are due to two actions: (1) The peropheral stimulation through the natural nervous reflexes inducing greater activity in the deep centers, whereby the respiratory excursions are deepened and the circulation of the blood accelerated, and (2) increased activity of the emunctories, particularly evidenced in the increased activity of the vegetative functions.

The local effects are (1) the induction of local hyperemia with local tissue relaxation, and (2) the secondary effects of hyperemia increased nutrition, increased local metabolism and increased phagocytosis. Radiant light and heat are of remarkable value for the treatment of all forms of infection, as effected by both the greater and lesser frequencies.

The clinical and biological changes in the body's fluids are due to (1) the results of increased elimination and local effects upon bacteria of the ultra-violet visible and infra-red rays, and (2) the consequent relief from toxic conditions, and (3) the action of the rays upon the contents of the blood stream. In anemia the ultra-violet rays and the luminous rays have been observed to increase the percentage of hemaglobin with marked increase of the red blood cells as demonstrated in the clinical changes in the blood findings of patients under treatment.

*Therapeutic Indications:* In conditions of impaired metabolism, where early inflammation is present without stasis, it is possible by the increase in active hyperemia induced to dissipate such conditions. Radiant energy is far more effective in these conditions than local applications of heat by hot fomentations of dry heat, because the effect is to produce heat deeper in the tissues, while to the surface there is applied as great an amount of heat as the tissues can tolerate.

The derivative effects of prolonged general applications of radiant light and heat are also markedly beneficial by producing an active superficial hyperemia. Congestion of internal organs is thereby in some degree relieved, and at the same time there is a lowering of arterial tension with increased elimination through the emunctories.

From the foregoing it will be readily seen that there exists a vast field of therapeutic possibilities in radiant light and heat.

In the infections conditions alone a great field is opened for type of physical energy, pyogenic infection being especially susceptible to the forms of radiant energy.

Abscesses, including boils and carbuncles, are remarkably affected, and in early stages are promptly absorbed by such treatment. In otitis media and mastoiditis, as demonstrated and published first by Dr. Herbert F. Pitcher, radiant light and heat from an incandescent source plays a most important part. In chronic otitis media, as well as in the acute forms, the remedy is practically specific. Prolonged applications are most serviceable in the treatment of erysipelas and gonorrheal ophthalmia and other infections susceptible to light and heat.

In conditions of impaired metabolism following exhausting diseases and associated with chronic infectious processes, radiant light from bath or large incandescent light applicators is invaluable. By stimulation of elimination by the skin and kidneys the functions are accelerated.

In secondary anemias the ultra-violet rays play an important part in increasing the percentages of hemoglobin and red cells in the blood.

Another very useful administration of radiant light and heat is the post-operative application over the site of surgical operation, when it hastens repair, prevents infections and lessens scarring by favoring active circulation.

In various skin diseases, as erythema, urticaria and varicose ulcers, treatment by radiant light and heat gives remarkable results. Another useful field is in rhus poisoning and for the control of X-ray dermatitis, in which last is a specific when applied before skin necrosis. The visible and infra-red rays produce stimulating heat and other effects, as outlined, while the characteristic effect of the X-ray, except with very short exposures, is inhibitory, arresting the functions of the tissues exposed, determining a local tissue inertia, and in large doses the death of the normal cells. In other words, they lessen the functional activities, both normal and abnormal. Reflected radiant light and heat is an exact antithesis to the X-ray, stimulating and restoring the parts so affected when timely long exposures are instituted and persisted in as indicated.

